# Assessment of peri-implant tissue dimensions following surgical therapy of advanced ligature-induced peri-implantitis defects

**DOI:** 10.1186/s40729-020-00282-y

**Published:** 2021-01-11

**Authors:** Ausra Ramanauskaite, Frank Schwarz, Robert Sader, Jürgen Becker, Karina Obreja

**Affiliations:** 1 Department of Oral Surgery and Implantology, Johann Wolfgang Goethe-University Frankfurt, Carolinum, 60596 Frankfurt am Main, Germany; 2 Department for Oral, Cranio-Maxillofacial and Facial Plastic Surgery, Medical Center of the Goethe University Frankfurt, Frankfurt am Main, Germany; 3 Department of Oral Surgery, Westdeutsche Kieferklinik, Universitätsklinikum Düsseldorf, 40225 Düsseldorf, Germany

**Keywords:** Animal experiments, Peri-implantitis treatment, Bone regeneration, Dental implant soft tissue

## Abstract

**Background:**

To evaluate peri-implant tissue dimensions following implantoplasty and/or regenerative therapy of advanced ligature-induced peri-implantitis in dogs.

**Material and methods:**

At all defect sites (*n* = 6 dogs, *n* = 48 implants), the intrabony component was filled with a particulate bovine-derived natural bone mineral (NBM). The supracrestal component was treated by either the application of an equine bone block (EB) or implantoplasty. In a split-mouth design, NBM and EB were soak-loaded with rhBMP-2 or sterile saline. All sites were covered using a native collagen membrane and left to heal in a submerged position for 12 weeks. The horizontal mucosal thickness (hMT) and bone thickness (hBT) were measured at four reference points: (v0) at the level of implant shoulder (IS), (v1) 50% of the distance IS-bone crest (BC), (v2) at the BC, and (v3) at the most coronal extension of the bone-to-implant contact.

**Results:**

The general tendency indicated a gradual increase in hMT from the IS (v0) toward BC (v2), which was more pronounced at implant sites treated with the regenerative approach. The hBT values increased from v2 to v3, with the highest values at the v3 region measured for implant sites treated with adjunctive rhBMP-2. For sites treated with implantoplasty, the linear regression model demonstrated an inverse correlation between hMT and hBT, whereas a positive correlation was observed at those sites treated with the regenerative approach.

**Conclusion:**

Horizontal soft and hard tissue dimensions were similar among different treatment groups.

## Introduction

Due to a high prevalence, the management of peri-implantitis has become a common issue in daily clinical practice [1]. Over the past few decades, numerous protocols have been proposed to arrest disease progression. While non-surgical approaches have shown limited clinical outcomes, surgical techniques with or without adjunctive resective and/or augmentative measures tended to be associated with a higher efficacy in reducing bleeding on probing and probing depths [2]. On the other hand, however, surgical procedures were commonly associated with the occurrence of mucosal recessions [3–6]. In fact, the assessment of the changes in peri-implant soft tissue level following therapy has recently been defined as an important clinical outcome measure [7], since it may affect the overall esthetics following therapy.

Even though adjunctive augmentative measures have been shown to enhance radiographic bone levels over non-augmentative techniques, they failed to reduce the postoperative occurrence of mucosal recessions (augmentative therapy 1.2 mm; non-augmentative therapy 1.9 mm; *p* = 0.31) [8, 9]. In order to overcome these limitations, a simultaneous thickening of the peri-implant mucosa by means of connective tissue grafts has been proposed and shown to compensate for postoperative soft-tissue shrinkage [10].

From the clinical perspective, horizontal peri-implant mucosal thickness, also called biotype, was suggested to affect the occurrence of soft-tissue recession. In particular, at healthy implant sites, a thick tissue biotype has been associated with a lower frequency of facial soft-tissue recession over time when compared with sites exhibiting a thin biotype [11, 12]. Due to the inflammatory lesion and associated increase in mucosal thickness, the assessment of the biotype is challenging at peri-implantitis sites [13]. Accordingly, it appears to be reasonable that the aforementioned changes in peri-implant soft tissue level following surgical therapy of peri-implantitis may be mainly due to the resolution of the inflammatory lesion rather than a trauma caused by the intervention. On the contrary, however, preclinical and clinical data provide some evidence that the horizontal mucosal thickness and bone thickness were inversely correlated at healthy implant sites (i.e., the thicker the bone, the thinner the soft tissue) [14, 15].

At the time being, the influence of surgical treatment procedures on the horizontal dimensions of peri-implant tissues has not been considered in the available literature. In this context, it might be hypothesized that a surgical procedure which is intended to re-establish supracrestal bony support may be associated with a different dimensional structure of peri-implant tissues than non-regenerative procedures.

Therefore, this analysis aimed at histologically assessing horizontal peri-implant tissue dimensions following various surgical treatment approaches at experimentally induced peri-implantitis lesions in the canine.

## Material and methods

### Study design and animals

This article is a secondary analysis and reports on histological analysis of tissue biopsies obtained from a previous experimental study employing a total of six beagle dogs, aged 14–16 months (mean weight 31.6 ± 2.9 kg) [16]. During the experiment, the animals were fed once per day with soft-food diet and water ad libitum. The study protocol was approved by the appropriate local authority (Landesamt für Natur und Verbraucherschutz, Recklinghausen, Germany) and the current reporting followed the ARRIVE Guidelines.

### Anesthesia protocol and experimental procedures

The anesthesia/analgesia protocols and experimental procedures have been reported in detail previously [16].

In brief, following intramuscular sedation using 0.17 mg/kg acepromazine (Vetranquil 1%, Ceva Tiergesundheit, Düsseldorf, Germany), anesthesia was initiated using 21.5 mg/kg thiopental-sodium (Trapanal 2.5%, Altana GmbH, Konstanz, Germany). Inhalation anaesthesia was maintained by oxygen, nitrous oxide, and isoflurane. For intraoperative analgesia, 0.4 mg/kg piritramid (Dipidolor®, Janssen-Cilag GmbH, Neuss, Germany) and 4.5 mg/kg carprofene (Rimadyl®, Pfitzer Pharma GmbH, Karlsruhe, Germany) was administered by intravenous injection. For postoperative analgesia, piritramid and carprofene were applied subcutaneously for three days.

The experimental phases included the following procedures:

- *Phase 1 (tooth extraction)*: P1-M2 were gently removed bilaterally in the upper and lower jaws after tooth separation. The sites were allowed to heal for 10 weeks.
- *Phase 2 (implant placement)*: following the elevation of mucoperiosteal flaps, titanium implants (∅ 3.8 mm, length 11 mm, Camlog® Screw-Line Implant, Promote® plus, Camlog Biotechnologies AG, Basel, Switzerland) were inserted bilaterally at a distance of 10 mm apart. According to the surgical protocol provided by the manufacturer, the implant shoulder (IS) was located in a slight supracrestal position (0.4 mm). All implants were connected with matching wide-body healing abutments (∅ 3.8 mm, height 4.0 mm, Camlog) and left to heal in a transmucosal position for 6 weeks [16].
- *Phase 3 (ligature-induced peri-implantitis)*: peri-implant mucosal inflammation was initiated by the submarginal placement of cotton ligatures (4-0) around the neck of each implant [17] and the plaque control regimen was terminated. The ligatures were exchanged once every 4 weeks and removed when a radiographic bone loss of 60% was achieved. Subsequently, a progression period of 4 weeks was initiated and supported by a renewal of the plaque control regimen [16].
- *Phase 4 (surgical treatment)*: all implant sites (*n* = 6 dogs, *n* = 48 implants) underwent access flap surgery (AFS), including granulation tissue removal and surface debridement/decontamination using plastic curets (Straumann® Dental Implant System) + cotton pellets soaked in sterile saline. At all of the implant sites (*n* = 48 implants), the intrabony defect component was filled homogeneously with a particulate bovine-derived natural bone mineral (NBM) (BioOss® spongiosa granules, particle size 0.25–1 mm, Geistlich, Wolhusen, Switzerland). The randomization process led to comparable mean values of horizontal bone extent in all groups prior to the treatment. Each supracrestal compartment was randomly allocated to receive either a size-adapted equine-derived bone block (EB) (Geistlich prototype block, width 10 mm, thickness 5 mm, height 10 mm Geistlich Pharma AG, Wolhusen, Switzerland) (*n* = 24 implants) or implantoplasty (P) (*n* = 24 implants) using diamond burrs (ZR Diamonds, Gebr. Brasseler GmbH & Co. KG, Lemgo, Germany) and Arkansas stones. Block randomizations were performed according to computer-generated protocols (RandList, DatInf, GmbH, Tübingen, Germany). EB blocks were applied over the exposed implants following preparation of a central core (∅ 3.8 mm) by using the conventional rotating pilot and twist drills matching the implant diameter in ascending order. In a split-mouth design, the NBM and EB were randomly soak-loaded with rhBMP-2 (0.77 mg/ml, InductOs®, Wyeth Pharma GmbH, Muenster, Germany) (*n* = 24 implants) or sterile saline for 15 min (*n* = 24 implants).

**Fig. 1:**
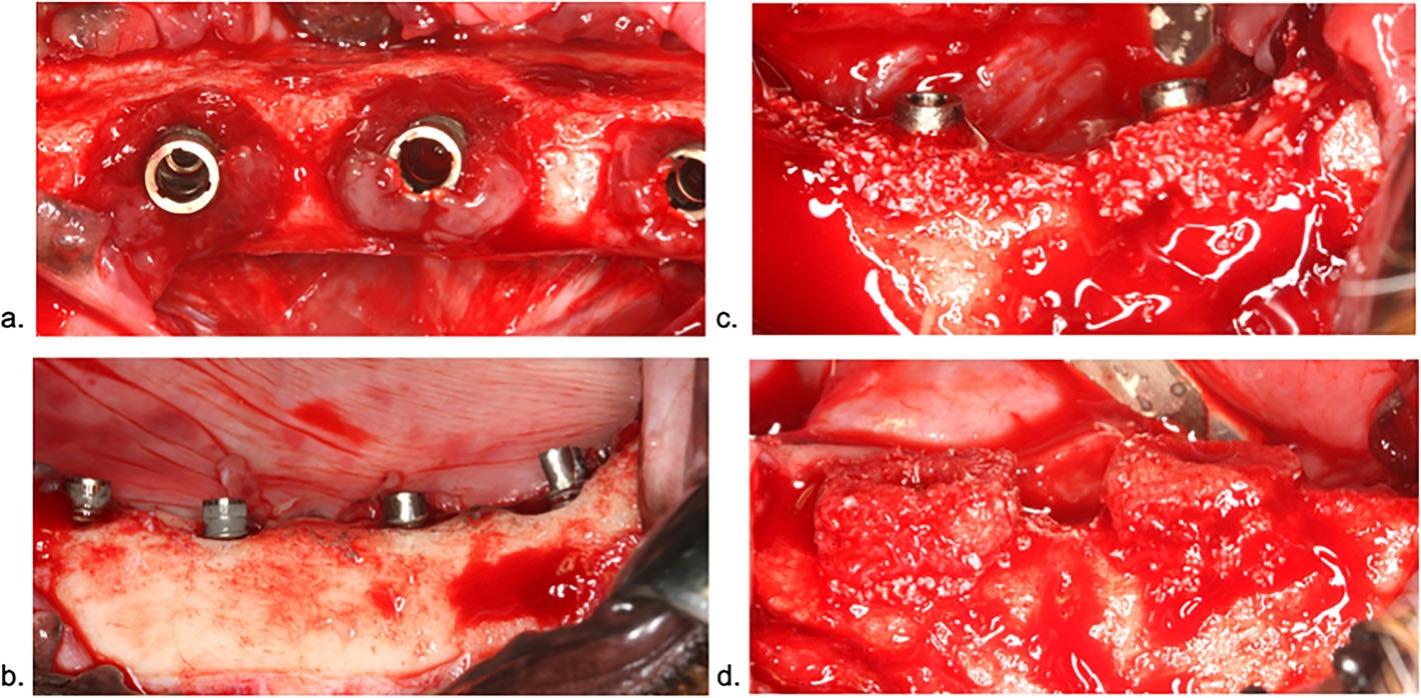
Surgical procedures. **a** Ligature-induced chronic-type peri-implantitis defects after phase 3. **b** Supra was randomly allocated to either the application of EB or implantoplasty (two implants at the right). **c** At all implant sites, the intrabony defect component was homogeneously filled with NBM. **d** EB blocks were applied over the exposed implants following preparation of a central core (Ø 3.8 mm). NBM and EB were randomly soak-loaded with rhBMP-2 or sterile saline

Following grafting, a native porcine-derived type I/III collagen membrane (CM) (BioGide®, Geistlich) was adapted over each defect site, covering 2–3 mm of the surrounding alveolar bone (Fig. 1). The mucoperiosteal flaps were repositioned coronally and fixed with vertical and horizontal mattress sutures to ensure a submerged healing procedure.

Accordingly, the following groups were tested in each animal (*n* = 6):

- ○ NBM (i)/EB + CM (2 defects) (referred to as EB)
- ○ NBM (i)/implantoplasty + CM (2 defects) (referred to as P)
- ○ NBM + rhBMP-2/EB + rhBMP-2 + CM (2 defects) (referred to as EB + rhBMP-2)
- ○ NBM + rhBMP-2/implantoplasty (s) + CM (2 defects) (referred to as P + rhBMP-2)

Wound healing was supported by the prophylactic administration of clindamycine (11.0 mg/kg body weight, Cleorobe®, Pharmacia Tiergesundheit) for 10 days.

### Histological preparation

The animals were euthanized (overdose of sodium pentobarbital 3%) after a healing period of 12 weeks, and the oral tissues were fixed by perfusion with 10% buffered formalin administered through the carotid arteries. The histological preparation has been reported in detail previously [16]. In brief, the tissue biopsies were dehydrated using ascending grades of alcohol and xylene, infiltrated and embedded in methylmethacrylate (MMA) (Technovit 9100 NEU, Heraeus Kulzer, Wehrheim, Germany) for non-decalcified sectioning (Exakt®, Apparatebau, Norderstedt, Germany). The most central section prepared at each implant site in the vestibulo-oral direction was ground to a final thickness of approximately 40 μm and stained with toluidine blue.

### Histological analysis

For the present analysis, only specimens exhibiting a fully intact and non-infiltrated soft tissue compartment (*n* = 36 specimens, EB: *n* = 9; *P*: *n* = 9; EB + rhBMP-2: *n* = 9; P + rhBMP-2: *n* = 9) were considered.

Digital images (original magnification × 200) were obtained from each specimen and evaluated using a software program (ImageJ 1.51s, National Institutes of Health, Bethesda, USA).

**Fig. 2:**
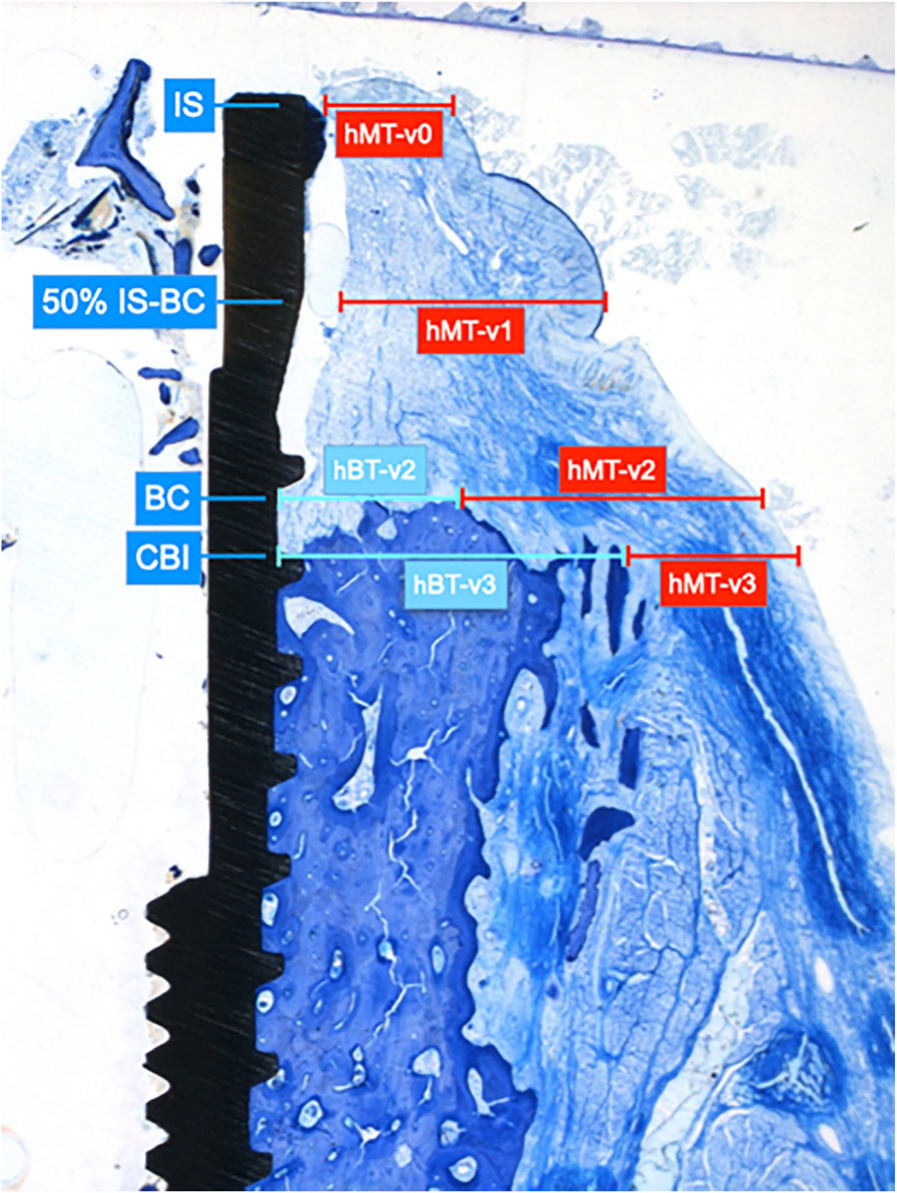
The following reference points served for the assessment of hMT (red lines) and hBT (turquois lines) values (specimen of the EB group): IS: implant shoulder, BC: bone crest, 50% IS-BC, CBI coronal extension of bone-to-implant contact

The following landmarks were identified at the vestibular aspect of each implant site (Fig. 2): IS, the bone crest (BC), the most coronal extension of the bone-to-implant contact (CBI). The horizontal mucosal thickness (hMT) and bone thickness (hBT) was measured perpendicularly to the implant axis at the level of IS (v0), 50% IS-BC (v1), BC (v2), and CBI (v3).

All histomorphometrical measurements were performed by one investigator masked to the specific experimental conditions. Intra-examiner calibration was performed using 10 histological sections that were evaluated on two occasions, 48 h. The calculated mean variability between the repeated measurements was 0.95 ± 0.04.

### Statistical analysis

The statistical analysis of the data sets was accomplished using a commercially available software program (IBM SPSS Statistics 26.0, IBM Corp., Armonk, NY, USA).

Mean values, standard deviations, and medians were calculated for all parameters in different groups defining the animal as statistical unit. The data rows were examined with the Shapiro-Wilk test for normal distribution. Between group comparisons were accomplished using the Mann-Whitney *U* test. Linear regression models were calculated to evaluate the dependence between hMT and hBT values. Landmarks at which either no BT or MT was present in the horizontal direction were excluded from the analyses. Results were considered statistically significant at *p* < 0.05.

## Results

**Fig. 3:**
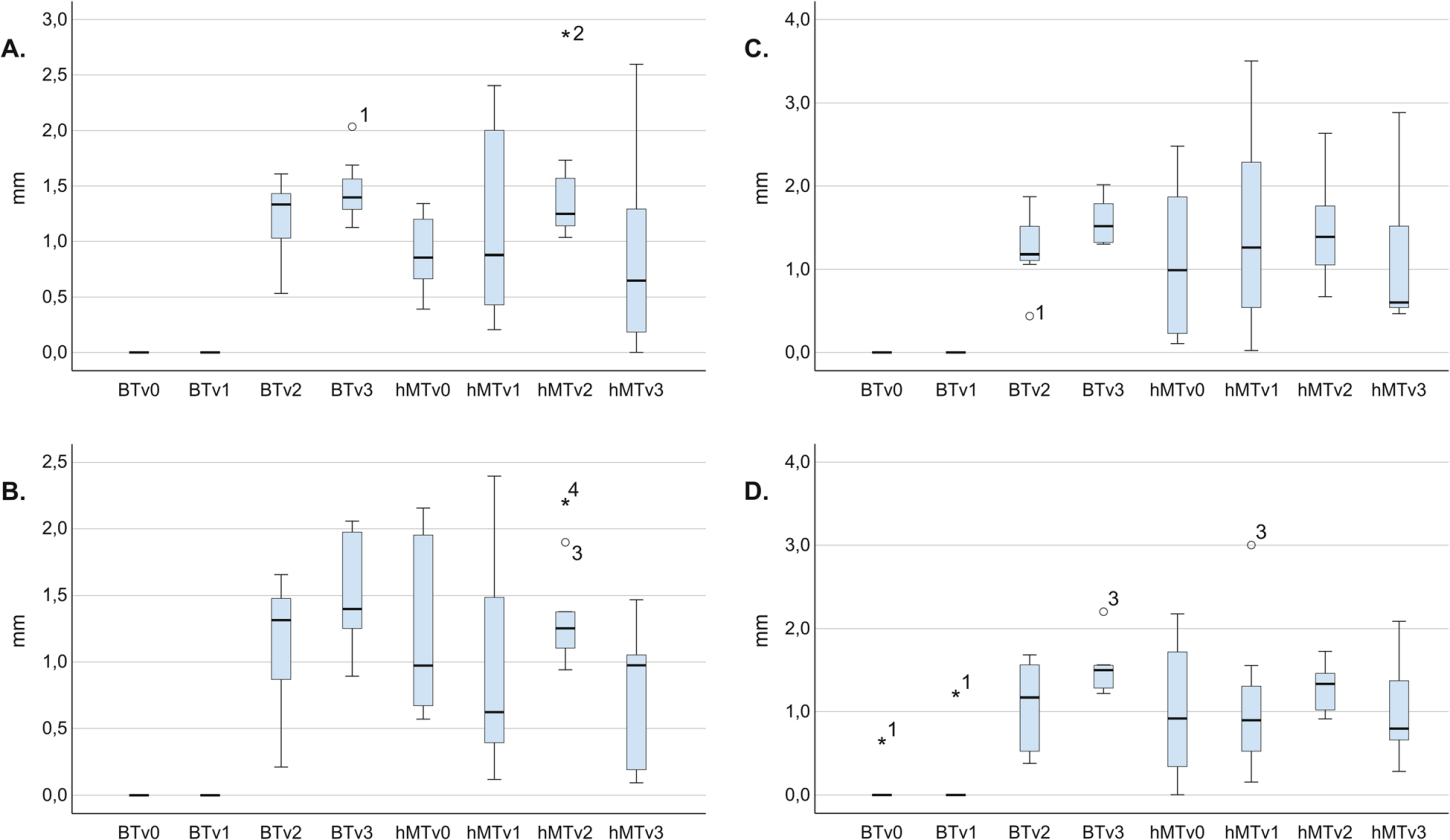
Boxplots indicating hMT and hBT values in different groups. **a** EB. **b** P. **c** EB + rhBMP-2. **d** P + rhBMP-2

Box plots indicating the hMT and hBT values at reference points v0–v3 in different treatment groups are presented in Fig. 3a–d.

At the implant shoulder (v0), hMT ranged from 1.204 mm to 0.899 mm, with the highest values measured in the P group and the lowest noted for the implants in the EB group, respectively. Except for the P treatment group, there was a tendency toward a gradual hMT increase from the implant shoulder (v0) toward the bone crest (v2) and a marked decrease from the v2 to v3 reference points. In the P group, the general trend indicated a thickness decrease from v0 toward v1, which was followed by an increase from v2 toward the v3 reference point. At the v1 and v2 reference points, the hMT values were higher at implant sites treated with regenerative therapy. With respect to the hMT changes from v0 to v2, the greatest increases were registered in the EB (0.583 mm) and EB + rhBMP-2 (0.37 mm) groups. Consequently, the highest hMT values in all groups were registered in the v2 region, particularly favoring sites treated with the regenerative approach (EB: 1.482 mm and EB + rhBMP-2: 1.468 mm vs. P: 1.354 mm and P + rhBMP-2 1.287 mm). The EB group yielded the most pronounced thickness decrease from v2 toward v3 (0.621 mm), followed by the P group (0.566 mm), whereas a lower hMT reduction occurred at implant sites treated with adjunctive rhBMP-2 (P + rhBMP-2: 0.302 mm; EB-BMP-2: 0.379 mm).

In the v2 region, higher hBT values were present at implant sites treated with the regenerative approach (EB: 1.218 mm; EB + rhBMP-2: 1.240 mm) compared to those treated with implantoplasty (P: 1.144 mm; P + rhBMP-2: 1.091 mm). In all treatment groups, the general tendency indicated an hBT increase from the v2 to v3 reference points. In particular, a higher increase in mean hBT values was found at implant sites in the P (0.355 mm) and P + rhBMP-2 (0.421 mm) groups in comparison to those referring to EB (0.243 mm) and EB+rhBMP-2 (0.33 mm) groups. The greatest hBT values in the v3 region were yielded at the implant sites treated with adjunctive rhBMP-2 (P + rhBMP-2: 1.512 vs. P: 1.499 mm; EB + rhBMP-2: 1.573 mm vs. EB: 1.461 mm).

**Fig. 4:**
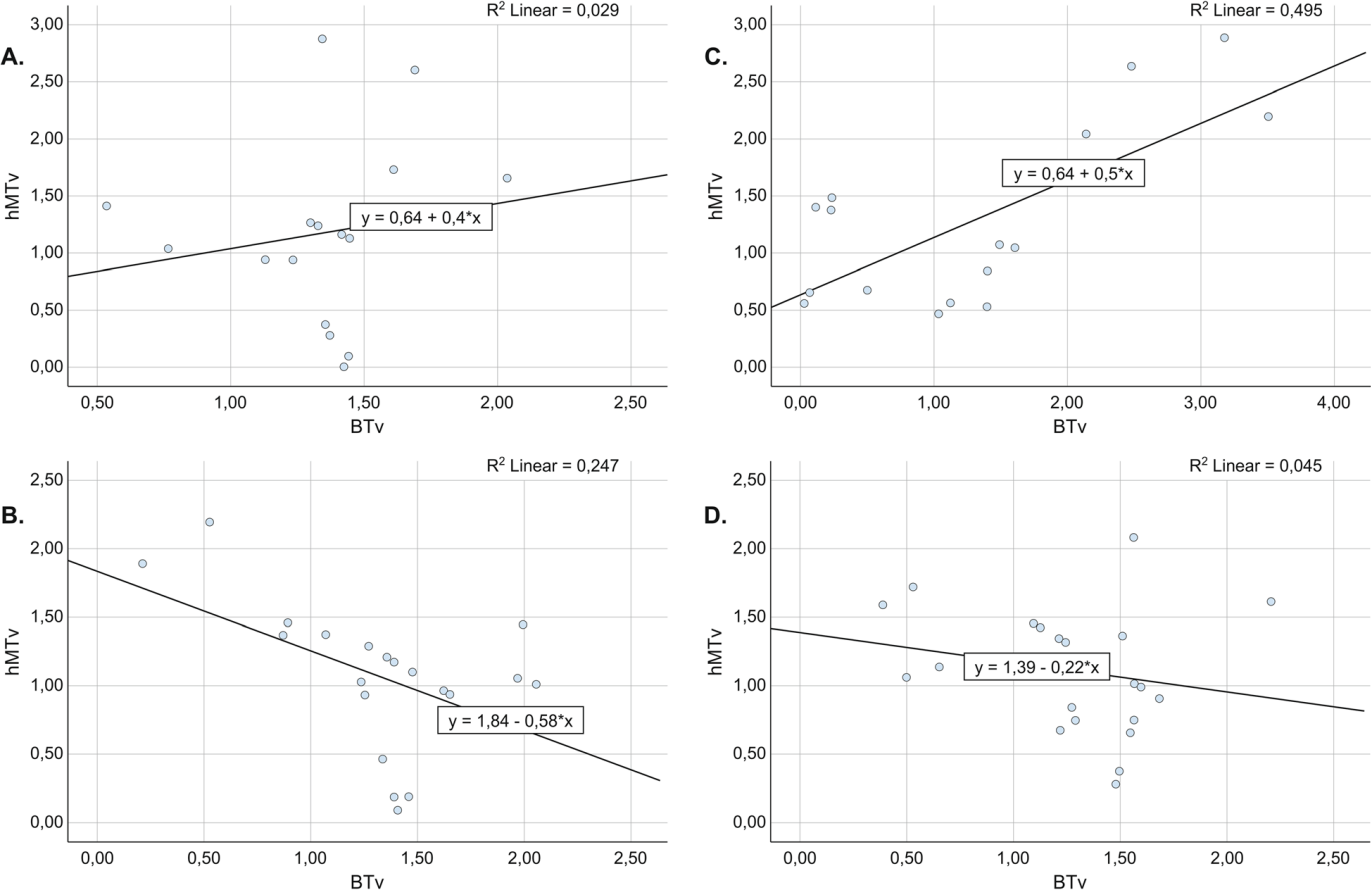
Regression plots depicting the dependency between mucosal thickness (hMT) and bone thickness (hBT) values at v0-v3. **a E**B (*R*^2^ = 0.029, *B* = 0.396, *p* = 0.532). **b** P (*R*^2^ = 0.247, *B* = − 0.426, *p* = 0.026). **c** EB + rhBMP-2 (*R*^2^ = 0.495, *B* = 0.50, *p* = 0.002). **d** P + rhBMP-2 (*R*^2^ = 0.045, *B* = − 0.217, *p* = 0.345)

Regression plots depicting the dependency between hMT and hBT are presented in Fig. 4a–d. The linear regression model showed an inverse correlation between hBT and hMT at implant sites treated with implantoplasty, which reached a statistically significant level in the P group (P: *R*^2^ = 0.247; *B* = − 0.426; *p* = 0.026; P + rhBMP + 2: *R*^2^ = 0.045; B = − 0.217; *p* = 0.354). In contrast, at sites treated with the regenerative approach, a positive correlation between hMT and hMT was detected, which was statistically significant in the EB + rhBMP-2 group (EB: *R*^2^ = 0.029; *B* = 0.396; *p* = 0.532; EB + rhBMP-2: *R*^2^ = 0.495; *B* = 0.50; *p* = 0.002).

## Discussion

The aim of the present analysis was to assess horizontal peri-implant tissue dimensions following implantoplasty and/or regenerative treatment approaches for the supracrestal defect compartments at experimentally induced peri-implantitis lesions in a canine model. All of the lesions featured advanced peri-implant bone resorption with a combined defect configuration including supracrestal and intrabony compartments, which the original study reported in detail [16].

Based on the histological data, both treatment concepts showed a tendency toward consistent increases in horizontal soft-tissue thickness from the implant shoulder to the bone crest, which aligns with the results of a previous preclinical study [14]. Notably, the mean hMT assessed at the implant shoulder was lower than that previously reported for healthy implant sites exhibiting dehiscence-type vestibular defects in the canine (range 0.899 mm to 1.204 mm versus 1.6 mm, respectively) [14]. With respect to the treatment approach, higher hMT was present at all investigated reference points. Additionally, mean MT in the apical direction tended to increase more at implant sites where supracrestal defect components were treated with regenerative measures. To the best of our knowledge, no data currently report on soft-tissue dimensions at peri-implantitis sites following different surgical treatment strategies.

In regard to the hard-tissue dimensions, hBT in the crestal area was higher at implant sites treated with a regenerative approach than at sites treated by implantoplasty (EB 1.22 mm; EB + rhBMP-2 1.24 mm; P 1.14 mm; P + rh-BMP-2 1.09 mm), but without reaching a significant difference. In all treatment groups, a general trend pointed toward BT increasing in the apical direction (i.e., toward the v3 reference point), with the highest hBT noted for sites treated with adjunctive rhBMP-2. This observation supports the findings of prior preclinical studies supporting the biological capacity of rhBMP-2 to promote osteogenesis and subsequently enhance bone regeneration [18, 19]. Furthermore, considering the treatment of peri-implantitis lesions, the present findings corroborate those reported in one previous experimental study in monkeys, where advanced peri-implantitis intrabony defects treated with adjunctive rhBMP-2 administered via a collagen sponge resulted in significantly higher vertical bone gain and re-osseointegration rates in the defect areas, as compared to the control sites (test 2.6 ± 1.2 mm and 29 ± 10.5%; control 0.8 ± 0.8 mm and 3.5 ± 2.5%, respectively) [20]. The beneficial effects of rhBMP-2 on bone formation and re-osseointegration in peri-implantitis defects were later supported by histological analyses in canine models, which demonstrated a tendency toward higher bone regeneration and re-osseointegration at peri-implantitis sites treated with adjunctive rhMBP-2 compared to those at the control sites, although the noted differences were not statistically significant [16, 21].

Further analysis of the present data revealed an inverse correlation between the horizontal thickness of soft and hard tissues at sites treated with implantoplasty, thereby indicating that an increase in hBT was associated with decreased mHT values. The aforementioned association was weaker at sites treated with rhBMP-2, which might be attributed to the enhanced peri-implant tissue dimensions in the corresponding treatment group (i.e., the P + rhBMP-2 group). The observed correlation essentially agrees with the findings of previous clinical and preclinical analyses. In particular, an experimental study in dogs demonstrated an increase in mucosal thickness in the presence of facial peri-implant bone deficiencies, with the highest MT measured in the absence of a facial bone plate [14]. In addition, a negative correlation between horizontal soft- and hard-tissue thicknesses at the IS was recently confirmed in a clinical setting [15]. The opposite association was observed at sites treated with regenerative measures at the supracrestal defect compartments, pointing toward increased BT being associated with increased hMT. In contrast to resective treatment approaches, the positive correlation was particularly true for sites where adjunctive rhBMP-2 was applied, thus once again pointing to the beneficial effect of rhBMP-2 on the horizontal dimensions of peri-implant tissue [19].

In this context, it is worth noting that the histological analysis showed that the sites treated with implantoplasty (i.e., P and P + rhBMP-2) commonly presented with a slight to moderate deposition of titanium particles in the adjacent tissues [16]. These were associated with localized chronic inflammatory infiltrate that appeared to be encapsulated by fibrous connective tissue. However, as suggested by a recent analysis of existing preclinical in vivo and clinical data, implantoplasty does not seem to be associated with any remarkable mechanical or biological complications in the short- to medium-term [22].

The results of the present analysis suggest that, in terms of horizontal peri-implant tissue dimensions, the use of regenerative measures for treating the suprabony components at peri-implantitis sites may lead to enhanced peri-implant dimensions compared to non-regenerative approach (i.e., implantoplasty). Therefore, one might assume that in the clinical setting, a regenerative therapy would help maintain postoperative tissue dimensions, leading to improved soft-tissue levels. Nevertheless, a previous analysis of the present data set revealed a higher exposure rate among the regenerative materials during postoperative healing (6 sites), thus showing that non-regenerative therapy (i.e., P or P + rhBMP-2; 3 sites) may offer a better ratio of defect resolution to potential susceptibility to reinfection of the exposed surface areas than a regenerative approach [16]. Considering the aforementioned observations, the clinical relevance of the present findings needs to be further elaborated.

The present analysis lacked a true negative control group (i.e., untreated rough implant surface at the supracrestal defect compartment); thus, we were unable to clarify the true possible beneficial effect of adjunctive treatment measures (i.e., implantoplasty or a regenerative approach) for the horizontal soft and hard tissue dimensions. In addition, the outcomes of the present analysis could not be compared to those that might have been obtained from using autogenous bone to reconstruct horizontal defects. Nonetheless, the available clinical data point to a lower radiographic defect fill and improvements in bleeding on probing following application of particulate autogenous bone when compared with bovine bone for the reconstructive therapy of peri-implantitis [22, 23]. This might possibly be related to the faster resorption of autogenous bone [24], which is further enhanced by the inflammatory conditions occurring in the adjacent tissues [7]. Finally, it is noteworthy that the absence of preoperative assessment of soft tissue biotype (i.e., prior induction of the disease) did not allow us to elucidate the possible influence of initial soft tissue thickness on dimensional tissue outcomes following the different surgical treatment approaches.

Within its limitations, the present analysis has pointed to similar horizontal soft and hard tissue dimensions among different treatment groups.

## Data Availability

Not applicable.
